# HIV Disclosure to Partners and Family among Women Enrolled in Prevention of Mother to Child Transmission of HIV Program: Implications for Infant Feeding in Poor Resourced Communities in South Africa

**DOI:** 10.5539/gjhs.v5n4p1

**Published:** 2013-03-07

**Authors:** Sphiwe Madiba, Rosemary Letsoalo

**Affiliations:** 1 Department of Environmental and Occupational Health, School of Public Health, University of Limpopo, Medunsa Campus, South Africa

**Keywords:** disclosure, mixed feeding, PMTCT, exclusive breastfeeding, exclusive formula feeding, postnatal, HIV-positive mothers, partners, family

## Abstract

The introduction of routine HIV counselling and testing (HCT) has increased the number of pregnant women being tested and receiving prevention of mother to child transmission of HIV (PMTCT) interventions in South Africa. While many women may enroll in PMTCT, there are barriers that hinder the success of PMTCT programmes. The success of the PMTCT is dependent on the optimal utilization of PMTCT interventions which require the support of the woman's partner, and other members of her family. We conducted focus groups interviews with 25 HIV-positive post-natal women enrolled in PMTCT, in the City of Tshwane, South Africa. The study explored HIV-positive status disclosure to partners and significant family members and assessed the effect of nondisclosure on exclusive infant feeding. Most women disclosed to partners while few disclosed to significant family members. Most women initiated mixed feeding practices as early as one month and reported that they were pressurized by the family to mix feed. Mixed feeding was common among women who had not disclosed their HIV-positive status to families, and women who had limited understanding of mother to child transmission of HIV. Women who disclosed to partners and family were supported to adhere to the feeding option of choice. Health providers have a critical role to play in developing interventions to support HIV pregnant women to disclose in order to avoid mixed feeding. Improving the quality of information provided to HIV-positive pregnant women during counselling will also reduce mixed feeding.

## 1. Introduction

South Africa initiated the National Prevention of Mother-to-Child Transmission of HIV (PMTCT) programme in 2001 to prevent the baby from acquiring HIV. Within ten years, interventions to prevent mother-to-child transmission (MTCT) of HIV are offered in more than 95% of public antenatal and maternity facilities country-wide (Goga, Dinh, Jackson, & group, 2012). In line with the 2009 WHO guidelines, PMTCT interventions in South Africa were modified and include routine HIV testing and counselling (HCT) for pregnant women, dual therapy to prevent MTCT, Highly Active Anti Retroviral Therapy for pregnant women with CD4 cell count ≤350 cells/μl, postnatal infant prophylaxis for breastfeeding HIV-positive women, and counseling for safer infant feeding practices (NDOH/SANAC, 2010). The routine HCT has increased the number of pregnant women being tested and receiving PMTCT interventions. Currently in South Africa, the uptake of PMTCT services is high, with more than 98% of women getting HIV tested during pregnancy and 92% of HIV-positive mothers receiving antiretroviral treatment or prophylaxis (Goga et al., 2012). Consequent to the implementation of the new guidelines, the national HIV transmission rate from mother-to-child, measured in infants aged 4-8 weeks dropped from 8% in 2008 to 3.5% in 2010. A further drop to 2.7% in the transmission rate from mother-to-child was recorded in 2011 (Goga et al., 2012).

While many women may enrol in a PMTCT programme, there are many barriers that impede the success of PMTCT programmes in many settings in sub Saharan Africa. One of the obstacles is the pregnant woman's challenge to disclose HIV-positive status to the sexual partner (Medley, 2004). Studies indicate that partner disclosure is a major condition for the success of the PMTCT program (Moland et al., 2010; Njunga & Blystad, 2010; Rujumba et al., 2012). HIV status disclosure serves as an important prevention strategy in PMTCT and enhances adherence to key PMTCT interventions. Disclosure also leads to increased utilization of preventive strategies and allows an individual to get partner or family support for preventive actions they may decide to undertake (Mucheto et al., 2011; Stirratt et al., 2006). It has been documented that women are more likely to adhere to PMTCT interventions when they have the support of their sexual partners (Medley, Garcia-Moreno, McGill, & Maman, 2004). However, in order to receive support, the women must disclose their HIV-positive status (Stirratt et al., 2006). Women who hide their HIV status are less likely to adhere to PMTCT interventions and might put their children at risk because they may be unable to take Anti Retroviral Therapy (ART) until delivery, fail to give Nevirapine syrup to the infant for recommended duration, and fail to adhere to the exclusive infant feeding option they selected (Kebaabetswe, 2007; Olagbuji et al., 2011; Rujumba et al., 2012). The importance of disclosure in the prevention of MTCT of HIV makes it a fundamental topic for inclusion in counseling HIV pregnant women in the PMTCT program (Baek et al., 2009).

The 2001 WHO guidelines on infant feeding recommend exclusive breastfeeding (EBF) with early and rapid cessation at six months or exclusive formula feeding (EFF) with free infant formula provided until 6 months. Exclusive breastfeeding is the most suitable option for women to prevent MTCT when replacement feeding is not acceptable, feasible, affordable, sustainable and safe. According to the WHO, breastfeeding can be made safer if either the mother or the infant takes antiretroviral medication during the period of breastfeeding (World Health Organisation [WHO], 2009).

However, studies show that the recommended exclusive breastfeeding or formula feeding options within PMTCT are often difficult to adhere to (Leshabari, Blystad, & Moland, 2007; Moland et al., 2010). This is because both feeding options are alien concepts in African societies where mixed feeding is the norm (Laar & Govender, 2011; Magoni et al., 2005). Compared with EFF or EBF, mixed feeding has been shown to increase the risk of HIV transmission to the baby and is highly discouraged in the first 6 months (Coovadia et al., 2007; Desclaux & Alfieri, 2006). Although a woman makes the decision on how to feed her baby during or after counselling, in practice, she may not be able to carry out that decision. Women are influenced by family beliefs, cultural practices and social circumstances in their homes to adhere or depart from their original choice of feeding (Leshabari et al., 2007; Matji et al., 2010; Njunga & Blystad, 2010; Thairu, Pelto, Rollins, Bland, & Ntshangase, 2005). They introduce other liquids before six months because of pressures placed on them by their mothers, mother in-law or grandmothers. In addition, mixed feeding is often a consequence of lack of disclosure of HIV status to family members and sexual partners (Doherty, Chopra, Nkonki, Jackson, & Persson, 2006; Fadnes et al., 2010; Sibeko, Coutsoudis, & Gray-Donald, 2009).

Studies show that partner disclosure is a central concept in PMTCT programs (Njunga & Blystad, 2010). However, in the context of South Africa where majority of HIV-positive mothers are young single women who live in extended families, disclosure to the sexual partner alone is not an adequate condition for the success of PMTCT. Although the optimal utilization of PMTCT interventions requires the support of the woman's partner and other members of her family (Igwegbe & Ugboaja, 2010), in South Africa, the HIV positive pregnant woman is often considered to be the primary target of PMTCT interventions. For PMTCT to succeed, it is important to provide adequate information on PMTCT interventions to the general population, the family, the in-laws, and male partners of HIV-positive pregnant women. There is a need for more discussion and openness about HIV positive status within households to shape community norms on infant feeding. As a consequence, create an easier environment for HIV positive women to carry out their infant feeding choices (Doherty, Chopra, Nkonki, Jackson, & Greiner, 2006).

Considering the importance placed on HIV status disclosure for the success of PMTCT interventions, there are limited studies that examine HIV status disclosure in the context of PMTCT. This study explored HIV disclosure to sexual partners and significant family members among post-natal women enrolled in a PMTCT programme in the City of Tshwane, South Africa. We also examined whether or not disclosing to partners and family members influenced exclusive infant feeding. The study will provide insights on HIV positive pregnant women's experiences of HIV disclosure to partners and significant family members within the PMTCT programme. Understanding HIV disclosure in the context of PMTCT is critical in order to provide support to HIV-positive mothers to adhere to PMTCT interventions.

## 2. Methods and Materials

### 2.1 Study Design

The study was conducted with HIV positive post-natal women who were enrolled in a PMTCT program of a community health centre in the City of Tshwane, Gauteng province, South Africa. The community centre provides services to a great number of clients from three informal settlements as well as an urban township. Data were collected between November 2010 and February 2011.

### 2.2 Data Collection

The data collection was carried out by the authors who are trained in conducting focus groups (FG). A research assistant also trained in qualitative methods assisted with recruitment and moderation of the FGs. An open ended FG guide was used for the FG interviews. The guide was developed in English and translated into Setswana, a local language spoken by most participants in the study site. The translations of the guide was done by the second author and verified by the first author, both authors an conversant with the local language. Participants were recruited if they were HIV-positive mothers enrolled in the PMTCT program and their babies were between six weeks and 6 months. Participants were recruited during the routine immunization follow up of their babies. Recruitment was done in the mornings while they awaited their turn to be seen by the nurse. After information had been provided, participants who were interested in being a part of the study were selected for the FG interviews. Mothers with babies younger that six weeks, as well as mothers with babies older than six months were excluded from participating in the study. The FG interviews were conducted in Setswana and were audio recorded with the participants’ permission. All FG interviews were conducted in a private room within the community centre, and each lasted about one hour. Informed consent was obtained from participants prior to the start of the interview. After each FG interview, the women received some refreshments. Four FG interviews were conducted, and each had an average of seven participants with a total of 25 participants.

A brief self-administered tool was used to capture socio-demographic information of the participants at the end of the FG interviews. The questionnaire collected demographic information including age, level of education, marital status and employment status. Data was also collected on the time of HIV diagnosis, disclosure to sexual partners and to others, and age of their babies. The tool was translated into Setswana, and participants who could not read and write were assisted to complete the questionnaire.

### 2.3 Ethical Approval

Ethical approval for the study was obtained from the Medunsa Research Ethics Committee of the University of Limpopo. Permission to conduct the study was granted by the Metsweding Region Research Ethics Committee and from the management of Laudium Clinic. Informed written consent was obtained after the study was explained; participants were also informed about the voluntary nature of the study and ability to stop the interview at any time or not answer specific questions. Participants were also assured that nonparticipation would not compromise their health care at the centre. The researchers ensured confidentiality throughout data collection.

### 2.4 Data Analysis

FG interviews were audio-taped and transcribed verbatim in Setswana and translated into English by the authors. The transcripts were reviewed for accuracy by replaying each interview recording whilst reading and translating the transcripts by the authors; both are well conversant with English and Setswana. Thematic data analysis was conducted by the authors who read the transcripts independently and jointly reviewed emerging themes to reach consensus on the interpretation of the data. The analysis was guided by the themes already contained in the FG guide. Following multiple readings of transcripts, themes were further refined and sub-themes related to disclosure of HIV status to sexual partners and significant family members were identified. After reaching consensus on the definitions of the themes and sub themes that captured the essence of the participants’ experiences of disclosure, NVivo version 8 a qualitative data analysis (QDA) computer software package, was used in the application of themes to the remaining transcripts. The transcripts were recoded if a new code emerged or an existing code was revised. Themes that were consistent in terms of the process of disclosure became categories.

To ensure trustworthiness of the FG findings; we conducted FG interviews in the local language, recorded the FG interviews, held peer debriefing sessions after each FG interview to discuss emerging themes, transcribed transcripts verbatim in the local language, verified raw data during translation, and used a computer software for analysis (Creswell, 2007; Patton, 2002).

## 3. Findings

### 3.1 The Socio-Demographic Characteristics of the Women

The 25 post natal HIV positive women who participated in the focus group interviews were aged between 18 and 40 years. Nine of them knew their status before pregnancy while 16 tested HIV positive in the PMTCT program. Eighteen of the women were single while seven were married. Ten women were living in a cohabiting relationship with their partners, five were living with their husbands, eight were living in extended families, and two were living alone. Seventeen of them were from informal settlements, and eight were from urban areas. Fifteen were unemployed and five were on part time and fulltime employment respectively. Ten of the women had disclosed their HIV-positive status to partners only, eight had disclosed to partners and close family members, two had disclosed to close family members only while 15 women had not disclosed their HIV-positive status to family members. Nineteen of the women did not know their partners HIV status. Of the 25 women, six were exclusively breast feeding, six were exclusively formula feeding, and thirteen were mixed feeding.

### 3.2 Themes

Seven main themes emerged from the data analysis; opinions on PMTCT, disclosure for support, fear of stigma and rejection, protecting others, protecting HIV status, and adherence to PMTCT interventions.

#### 3.2.1 Opinions on PMTCT

Women participating in the FG interviews were asked about their opinions and their experiences of participating in the PMTCT programme. Most women experienced PMTCT positively, and the desire to protect their babies from HIV infection influenced their participation.

*The program has helped me a lot because I have learned many things. Also, PMTCT assisted me to know that it is also important to breastfeed your baby even if you are HIV positive (27yrs single mother)*.

*When I was taking treatment, I was thinking about the safety of the baby and also my wellbeing because I knew that the more I drink my treatment, the more chances of my baby being safe and I will be there to take care of my baby. I did not want to be weak and sick after giving birth or even the possibility of getting a sick baby (29yrs single mother)*.

3.2.1.1 Knowledge of MTCT Transmission

All women received counseling on MTCT of HIV and infant feeding options at enrolment in PMTCT. Most women demonstrated a good understanding of exclusive breast feeding and correctly mentioned the importance of taking AZT

*If you breast feed your baby for six months it prevents infecting the baby with HIV and that you do not have to give formula milk while you are breastfeeding the baby (38yrs single mother)*.

*They always mention that they give us AZT, so as to protect our unborn babies from being infected with HIV. The other thing is to protect the baby when you are breastfeeding (34yrs cohabitating mother)*.

The data show that where knowledge and understanding of MTCT was low, women delayed taking AZT.

*During counselling they told me about the ART drugs; it was a little bit difficult for me to drink them. I was a bit in denial to take them before. And they called me and explained to me, then after that I understood as they said the treatment is going to boost my life, and make my body strong (26yrs single mother)*.

3.2.1.2 Opinions on counselling

At the time of data collection, women in the PMTCT program received pre and post-test counselling as well as infant feeding counselling which was provided as group counselling and or one to one counselling. Most women perceived the counselling they received as good and reported that it made it easy for them to deal with their HIV status.

*I gave my baby only bottle feeding; I was helped by the counseling that was provided to us. At that time, they were encouraging people to give formula milk, rather than breast milk. They said that sometimes breastfeeding can make my child be HIV positive (26yrs single mother)*.

However, because the WHO recommendations on infant feeding were modified several times, counselling of infant feeding became confusing for both heath care providers and pregnant women. As a result, a few women reported that the counselling was not so good.

*You become confused because other nurses say breastfeeding is okay, whereas others say breastfeeding is not right. In our clinic, they were emphasizing breastfeeding and discouraging formula feeding (29yrs single mother)*.

*In my case, they were encouraging formula feeding. I feel pain because I am experiencing difficulties when I am giving my baby the bottle in front of people, because they keep asking me why I only give bottle feeding to the baby (26yrs single mother)*.

In some instances, health care providers were prescriptive and offered only one feeding option that was favored by the counselor. Consequently women were forced to choose the feeding option that was promoted at the time.

*I started with breastfeeding as I had my baby through caesarean section. At the hospital, they did not want formula feeding, and they forced me to breastfeed. I was breastfeeding my baby with a bleeding heart; wondering if I was going to infect the baby through breastfeeding. I stayed four days in the hospital and after discharge when I got home I bought formula milk (32yrs married mother)*.

*They ask me if I was breast feeding my baby and I said yes. A sister at the clinic told me that they do not want breastfeeding and that it was not allowed, and she said I must stop breastfeeding (29yrs single mother)*.

#### 3.2.2 Disclosure for Support

Women disclosed their HIV status to sexual partners, parents, siblings, and friends. Those who disclosed to family members did so for the purpose of receiving support to adhere to PMTCT.

*Only three people know my HIV status, which is my two aunts because when I started with ART one of them was my treatment buddy so that she can support me with taking of treatment (26yrs single mother)*.

The decision to disclose was well considered, and women disclosed to family members and friends they trusted. An additional consideration for disclosure was if the women perceived the family member as having an understanding of the HIV disease.

*At least when you tell a person who understands what HIV is all about that person cannot judge you and go around talking bad about you to other people (27yrs single mother)*.

*As long as I told my boyfriend, my mother and my siblings, that is all. If they tell, it is their problem, but I trust that they will not tell. I did not even tell my elder brother, only my mom and my two younger sisters, and they also kept quiet (33yrs married mother)*.

#### 3.2.3 Partner Reaction to Disclosure

Women who had disclosed experienced varying reactions from their partners. The data show that some women received no support from their partners while some were abandoned after the disclosure.

*They tested me and told me that I was HIV positive, I went home and came back with my husband, they tested him and he was HIV negative. Since that day he never came back home, he has another woman he is staying with now (43yrs married woman)*.

*I told him that I was pregnant, and HIV positive, he was happy that I was pregnant, but the issue of me being HIV positive, he did not take it so well. It was the last time I saw him, he left (31yrs single mother)*.

One other reaction from partners that was experienced by women was that the partner denied the HIV-positive test of the women.

*I told my partner, but he did not believe me; he said I was mad, and I am the only one who is sick, “Tsonga” people do not suffer from HIV. He said I was the one who brought AIDS to the family (30yrs single mother)*.

In contrast, the data show that some women received support from their sexual partners in caring for their infants and adhering to ART medications.

*I told him to go take a test because I tested positive, so that if we are both positive we can take medication together. He took the HIV test and tested negative. I cried at the hospital, and he comforted me on our way home and told me that I should not worry that all will be fine (31yrs single mother)*.

#### 3.2.4 Stigma and Rejection

The most common reasons for nondisclosure to family members were fear of social rejection and discrimination.

*The most important thing that makes us not to disclose our status is fear of being rejected. In my family we are five children, if I can tell them that I am HIV positive and you find that they do not have the knowledge and clear understanding of HIV, if they no longer treat me as they used to treat me or they reject me, I am going to feel sad because I do not have support from my family (31yrs single mother)*.

The data show that most women did not disclose to family members when they believed that the family members have negative views and perceptions about HIV positive people.

*My mother has problems with HIV positive people. I had a cousin who passed away she was HIV positive; my mother was talking negatively about her, and she said she cannot stay with her children because one of them is HIV positive. Sometimes my cousin's children would visit us; my mother would not touch or bath them and she literally isolated them (29yrs single mother)*.

*Eish! my mother has serious problems with people who are HIV positive, when I had minor ailments, she encouraged me to go to for HIV counseling and testing, and when I came back she asked for the result, I said I was HIV negative, and she will said ‘it's better, if you were HIV positive I was going to chase you out of my house (27yrs single mother)*.

#### 3.2.5 Protecting Others

One other reason for not disclosing to family members was the desire to protect elderly parents from the burden of the HIV diagnosis. Women also feared that disclosure will compromise their health.

*I did not tell my mother because of the way she talks about the HIV disease. I think that if I tell her, she will pass out; she is too weak. She still sees HIV as an extremely dangerous disease, and she feel people with this disease can die at any time. She is too sympathetic towards HIV positive people. I decided not to tell her. It is better for her not to know (31yrs single mother)*.

*My mother is an old person, she is almost 90 years old; I realized that I will just be giving her stress; she will think that I will die tomorrow. So I decided to leave her, I am avoiding giving her stress and other illnesses (37yrs single mother)*.

Women who did not disclose to their sexual partners found disclosure extremely difficult. They were fearful that their partners would react with violence to the disclosure or accuse them of being responsible for the infection.

*The main thing that is worrying me is how he is going to feel when I tell him. I’m scared to tell him because he is one person that will want to tell everybody about my status in his family that I’m the one who gave it to him (24yrs single mother)*.

*If I tell him, he might take all the responsibilities thinking it is him who brought it or maybe kill me or leave me or kill himself to be out of my life (29yrs single mother)*.

#### 3.2.6 Protecting the HIV Status

Non-disclosure was commonly used as a strategy for protecting self from stigma and rejection. However, participating in PMTCT subjected women to scrutiny and questioning from family members, partners and the community. Most women responded with a lie to all questioning.

*When my partner asked for the reason for not breastfeeding, I told him that it is because of the rash on my breasts (26yrs single mother)*.

*My grandmother asked me why I was not breastfeeding my baby and I told her that I was having problems with my breasts and I cannot breastfeed the baby (30yrs single mother)*.

*Every morning when I take my pills, I also give my baby Nevirapine syrup. When they asked me why my baby was drinking medication every day I told them that she was having blocked nose (31yrs single mother)*.

The data show that women who feared disclosure devised strategies to continue hiding their HIV status from partners, families, and the community.

*I do not know what people think about the milk that we get free from the clinic (pelargon). I do not know if people in the community think that pelargon is only for HIV positive people. Even now after buying or collecting milk from the clinic I remove the label (29yrs single mother)*.

*With my tablets, immediately after collecting them from the clinic, I empty them into sachets and I throw away the ARVs bottles so that if somebody finds me drinking tablets, they must not know what I am taking (26yrs single mother)*.

#### 3.2.7 Adherence to PMTCT Interventions

HIV status disclosure to the partner is a major condition for the success of the PMTCT program. For women who disclosed to sexual partners, adherence to PMTCT procedures was easy.

*My boyfriend is helping me, I give my baby Nevirapine syrup at 8h30 in the morning and 8h30 in the evening, at around 8h20, he will buzz me to remind me to give the baby Nevirapine (29yrs single mother)*.

In contrast, women who had not disclosed reported that non-disclosure was a challenge for participation in PMTCT. Though they wanted to adhere to PMTCT interventions, it was often a challenge because they had not disclosed to family members.

*It was a challenge because I was supposed to hide the medication I got from the clinic because of the people I am staying with. I am staying with my sister and she does not know that I am HIV positive, so I was supposed to hide my medication, but still at the same time I was supposed to drink my medication on time and to make sure that my sister doesn’t see them. That is why I say it was a challenge (31yrs single mother)*.

It was also difficult to adhere to feeding options if women didn’t disclose to family members. Mixed feeding was thus often a consequence of not disclosing HIV status to family members.

*I was staying alone but my aunts came to help me when I had the baby. They didn’t say that I have to give my baby food they repeatedly said that I was starving the baby. Eish…, I ended up giving my baby porridge before six months, but I didn’t give my baby any water I could not bring myself to give him water (26yrs single mother)*.

Conflicting messages regarding infant feeding offered by healthcare workers tended to confuse women and influenced adherence to infant feeding.

*I was expressing about two months while I was in hospital because I was not having enough milk. After I was discharged I stopped breastfeeding the baby and gave formula but I continued giving the baby Nevirapine (28yrs single mother)*.

The data show that women were eager to adhere to PMTCT interventions despite not disclosing to family members and or partners. The data show the great lengths to which women would go to avoid disclosure of their HIV status but maintain adherence.

*After having the baby I came to the clinic so that they could write me a letter that supports that my baby must be exclusively breastfed so that when I go back to work I will be able to express and they will give breast milk to my baby (29yrs single mother)*.

*I pour my baby's Nevirapine in a juice bottle and when they ask me why the baby's medication is in a juice bottle I tell them that, at the hospital they give us medication in juice bottles (26yrs single mother)*.

Women who had good understanding of the implication of infant feeding on the transmission of HIV were able to resist pressures from the family to mix feed.

*It was difficult, the family would force you to give the baby water or porridge, and if you tell them that at the clinic they discourage us to give the baby porridge and water, they said you listen to nurses and doctors more than us. For me it was not easy, it was difficult, because at the clinic I was told that if the baby can drink some other stuff, the baby is at risk of being infected with HIV. It was not easy, but I managed to hold on the PMTCT program, but six months was too long for me because sometimes I felt that I was starving my baby because the baby didn’t drink water (26yrs single mother)*.

However, - women who did not understand MTCT of HIV and the implications of infant feeding in MTCT introduced solids and other fluids before six months.

*I am giving my baby purity (Mother of 2 months old baby)*.

*I was breastfeeding the baby, but now I stopped and I am giving the baby Infacare (Mother of 2 months old baby)*.

*I was breastfeeding but my breasts are having no milk and the baby was crying too much, after two days I bought Pelargon and gave the baby because it was hungry and crying a lot. I tried to breastfeed, but it was difficult and my breasts were sore. I am breastfeeding for two to four days and other days I was giving pelargon (Mother of 1 month old baby)*.

## 4. Discussion

The study found that 16 out of 25 women tested for HIV in the PMTCT programme. In line with other studies, being pregnant and HIV positive was a traumatic and emotional experience for women who feared for the safety of their unborn babies. The emotional experience of a positive result when a woman is pregnant is compounded by the possibility of passing the virus on to the child (Baek et al., 2009; Doherty, Chopra, Nkonki, Jackson, & Persson, 2006). Most of the women had a good understanding of PMTCT interventions, and the desire to protect their unborn babies from HIV infection influenced their participation. By participating in the PMTCT program, they were preventing their babies from being infected with HIV. In addition, women were motivated to stay healthy to raise their babies by adhering to their ART drugs. Similar findings of women staying healthy to raise their babies were documented in other studies (Brickley et al., 2009; Brou et al., 2007; Kasenga, Hurtig, & Emmelin, 2010; Ross, Sawatphanit, Draucker, & Suwansujarid, 2007).

Most women described the counselling provided at enrolment in the PMTCT programme as good. While a few felt confused and unsure about the best infant feeding option because of the conflicting messages provided by health workers during counselling. A small number of women changed from exclusive breast feeding to exclusive formula feeding because they were forced to breast feed by nurses in the hospital. Similarly, Matji et al. (2010) found that women who changed to exclusive breastfeeding from their original intention of exclusive formula feeding were forced to breastfeed in hospital. The poor understanding of MTCT resulted in women delaying and not taking AZT at all during pregnancy. The data suggest that understanding MTCT was influenced by counselling, women who received group counselling reported a delay in taking AZT during pregnancy. It was only after one to one counselling was provided that the women understood the importance of AZT in preventing MTCT. Inadequate counselling particularly on PMTCT feeding options resulted in an under-use of PMTCT services among women in another study (Nguyen, Oosterhoff, Ngoc, Wright, & Hardon, 2008).

Similar to previous studies, most women (18 out of 25) disclosed their HIV status to sexual partners (Brou et al., 2007; Makin et al., 2008; Medley et al., 2004; Olagbuji et al., 2011). Studies show that women who report being married or in a stable cohabiting relationship disclose their HIV-positive status as compared to single women (Brou et al., 2007; Makin et al., 2008; Medley et al., 2004; Olagbuji et al., 2011). Our data support this finding; in this study seven of the 25 women who did not disclose to their sexual partners were single women aged between 18-30 years. Data from previous studies show that young age is a likely barrier to disclosure among HIV-positive women. Young women found disclosure difficult and feared that they would be abandoned by their partners following disclosure (Ross, Stidham, & Drew, 2011; Rujumba et al., 2012; Ssali et al., 2010). Married and cohabiting women may feel more confident than single women who may not be in a stable relationship to discuss HIV infection (Brou et al., 2007; Olagbuji et al., 2011). Although most women disclosed to sexual partners, very few women disclosed to significant family members. These women lived with extended family members who were not informed of the women's HIV status. Doherty, Greiner, et al. (2006) reported the same findings, in their study, most women disclosed to the father of the child who in most cases was not living with the woman. Recent data show that women living with extended families were two times more likely not to disclose their HIV status as compared to women living alone or with partners (Mucheto et al., 2011).

The decision to disclose in this study was well considered and selective (Mucheto et al., 2011). Women disclosed to family members if they perceived them as having a good understanding of the HIV disease. Women who perceived greater support from their families prior to their diagnosis were likely to disclose (Ross et al., 2011). Furthermore, our data confirm findings that women who disclose their HIV status do so motivated by a need for support to adhere to PMTCT interventions, particularly their feeding choices (Sibeko et al., 2009; Ssali et al., 2010; Varga, Sherman, & Jones, 2006). In this study and others, women were supported to adhere to PMTCT interventions if they disclosed to family members. Disclosure to family members made it easy for women to explain their infant-feeding practices (Doherty, Chopra, Nkonki, Jackson, & Persson, 2006; Fadnes et al., 2010).

Many studies have shown that contrary to the anticipated fear of negative reactions from the partner, many men have been found to be supportive of their partners’ participation in the PMTCT programme (Brou et al., 2007; Igwegbe & Ugboaja, 2010; Medley et al., 2004; Olagbuji et al., 2011; Theuring, Nchimbi, Jordan-Harder, & Harms, 2010). We also found that women who disclosed to husbands or steady cohabiting partners were supported to adhere to infant feeding, as well as the baby's ART medication. These women were adhering to exclusive infant feeding and other PMTCT interventions at the time of data collection. The findings must be viewed in the context where 13 of the 25 women initiated mix feeding before six months.

Many HIV-positive women still faced disclosure challenges in their respective communities, resulting in low disclosure in PMTCT (Fadnes et al., 2010). Nondisclosure was used as a strategy for protecting self from potential stigma and rejection and has been documented in other studies (Olagbuji et al., 2011; Ross et al., 2007). Women were fearful of disclosure because they felt that once their family learned about their HIV status they will be rejected. According to Brickley et al. (2009), societal stigmatizing attitudes penetrate family relationships resulting in discrimination within the family.

In line with other studies, women who did not disclose their HIV status to their partners were fearful of a violent reaction from their partners (Rujumba et al., 2012; Ssali et al., 2010). The fear of a violent reaction may be compounded by serodiscordance with the male partner being HIV negative (Sadoh & Sadoh, 2010). In this study, 19 women did not know the HIV status of their partners and were fearful that the partners might test HIV negative. Most male partners did not go for HIV testing following disclosure, they assumed that they would have the same result as their partners (Falnes et al., 2011; Rujumba et al., 2012). Women also associated disclosure to partners to the risks of discrimination, abandonment, rejection, and violence. Similar findings were documented in other studies (Medley et al., 2004; Mucheto et al., 2011; Ssali et al., 2010). Though previous data show low levels of violent reactions from partners following disclosure (Brou et al., 2007; Medley et al., 2004; Rujumba et al., 2012; Theuring et al., 2010), in this study, seven out of 25 women were abandoned by their partners. In a Zimbabwean study, women were divorced after they disclosed to their husbands (Njunga & Blystad, 2010). Information on the low levels of violent reactions from sexual partners should be made available to women during post-test counseling on partner disclosure (Igwegbe & Ugboaja, 2010).

Lack of disclosure to family members made adherence to infant-feeding difficult in this study and others (Doherty, Chopra, Nkonki, Jackson, & Greiner, 2006). Although women wanted to adhere, in the absence of disclosure, it was difficult to adhere to their feeding options. Our findings support previous studies highlighting that infant feeding becomes complex without the support obtained through HIV status disclosure by the woman (Varga et al., 2006). As mentioned, disclosure makes it easy for women to explain their feeding practices while lack of disclosure makes resistance to increasing pressure from family members to introduce other liquids difficult (Doherty, Chopra, Nkonki, Jackson, & Persson, 2006). The influence of family members on infant feeding should be viewed in relation to the vulnerable situation of the women (Doherty, Chopra, Nkonki, Jackson, & Greiner, 2006). In this study, of the 15 women who did not disclose to family members, most were young, single, unemployed and lived in extended families.

Similar to other studies, we found that lack of disclosure to family members increased mixed feeding practices (Doherty, Chopra, Nkonki, Jackson, & Greiner, 2006; Fadnes et al., 2010; Sibeko et al., 2009). More than half (13) of the women were pressured by family members to give the baby soft porridge and or water before six months. Similar finding were documented in other studies (Akarro, Deonisia, & Sichona, 2011; Doherty, Chopra, Nkonki, Jackson, & Greiner, 2006; Maru et al., 2009). According to Laar and Govender (Laar & Govender, 2011) entrenched family and social pressure and cultural norms compel HIV-positive mothers in developing countries to maintain mixed feeding. More so in African culture were mixed feeding patterns are highly acceptable and often the baby is given other fluids and semi solids during the first few weeks of life (Akarro et al., 2011; Laar & Govender, 2011; Leshabari et al., 2007; Matji et al., 2010; Thairu et al., 2005). Although the pregnant woman has been the main target for PMTCT interventions in many settings, (Akarro, Deonisia, & Sichona, 2011); more openness about HIV status within households to shape community norms on infant feeding is recommended (Doherty, Chopra, Nkonki, Jackson, & Greiner, 2006). In the current PMTCT programme, families do not understand PMTCT interventions, hence the expectations that the HIV positive women should mix feed. The involvement of men in PMTCT is the first step in refocusing PMTCT programmes. However, Falnes et al. (2011) found that the major obstacle for male participation was the definition and organization of the PMTCT programme as fundamentally female oriented.

As stated, women participated in PMTCT to protect their babies from being infected with HIV. We found that women who had a good understanding of MTCT were eager to adhere to PMTCT interventions despite non-disclosure to family members. They went to considerable lengths to avoid disclosure but maintained adherence to infant feeding options. Doherty, Chopra, Nkonki, Jackson, and Persson (2006) argue that women with knowledge of the importance of exclusive infant feeding are able to resist the pressure placed on them by family members. Our data supports this view; women found alternative ways to adhere to infant feeding despite the interference from family members. One way they protected their HIV status was to lie about the reasons for exclusive infant feeding as well as the reasons for taking medication. Previous studies reported that women who had not disclosed engaged in creative strategies to avoid disclosure (Desclaux & Alfieri, 2006; Doherty, Chopra, Nkonki, Jackson, & Persson, 2006; Sibeko et al., 2009).

At the time of data collection, women were given the option between EFF and EBF for six months. In this study and others, women who opted for EFF were questioned extensively about their feeding options as compared to EBF mothers (Doherty, Chopra, Nkonki, Jackson, & Greiner, 2006; Kebaabetswe, 2007; Maru et al., 2009). In countries where breastfeeding is the norm, formula feeding mothers were often suspected to be HIV-positive by their communities (Desclaux & Alfieri, 2006; Fadnes et al., 2010; Laar & Govender, 2011). As a result, HIV positive women feel compelled to hide the fact that they formula feed their babies (Doherty, Chopra, Nkonki, Jackson, & Greiner, 2006; Laar & Govender, 2011; Matji et al., 2010). Women in this study and others used lies, deception, and ill health such as breast cancer and sore or itchy breasts to respond to questions on formula feeding (De Paoli, Manongi, & Klepp, 2004; Maru et al., 2009; Matji et al., 2010; Ware, Wyatt, & Tugenberg, 2006). As mentioned, women went to considerable lengths to protect their HIV status in this study. To conceal the fact that they were taking ART they used paracetamol or vitamin sachets for their ART medication, they used juice bottles to store nevirapine syrup, and they would tear off the cover from the milk they collect from the clinic. In a previous study conducted in South Africa, HIV positive mothers used similar strategies to hide their HIV diagnosis (Doherty, Chopra, Nkonki, Jackson, & Greiner, 2006).

In addition to the influence of the family on infant feeding, literature shows that PMTCT counselling influence adherence to infant feeding. Data show that a single counseling session does not provide adequate information to inform infant feeding decisions made by HIV positive women (Baek et al., 2009; Matji et al., 2010). According to Baek et al. (2009) women tend to remember more when they had received more than one counseling session. While a single counselling session is characterized by mixed messages and often results in mixed feeding (Matji et al., 2010). In this study, women felt confused and unsure about the best infant feeding option because health workers provided different messages. Consequently, women had limited understanding of HIV transmission risk through mixed feeding practices (Doherty, Chopra, Nkonki, Jackson, & Greiner, 2006; Doherty, Chopra, Nkonki, Jackson, & Persson, 2006; Sibeko et al., 2009). The findings suggest that good infant feeding counselling and support provided by health care workers could improve adherence to infant feeding.

The study has a number of limitations. The sample size is small and does not reflect the experiences of all HIV-positive women; therefore, our findings may not be generalizable to wider populations of HIV-positive women. The selection of participants was purposive and is subject to selection bias. Only participants who volunteered to participate in the study were recruited and the researchers did not document reasons for refusal to participate in the study. However, we recruited a wide selection of HIV positive women representing the population of women enrolled in PMTCT programmes in similar settings. Our findings are limited in that the women's reports of disclosure to partners and family may have been affected by social desirability. Secondly, our findings are assessed based on self-reports by the woman and may have resulted in recall bias. The study obtained information from women only and could not verify if they had indeed disclosed to partners and significant family members. Nevertheless, the data have relevance for PMTCT programmes in similar settings.

## 5. Conclusion

This study set out to explore whether HIV disclosure to partners and family members affected adherence to exclusive infant feeding. We found that adherence to exclusive infant feeding was influenced by factors within the social and cultural contexts of the women. For young HIV-positive mothers who live in extended families, disclosure is crucial for adherence to exclusive infant feeding. We found that mixed feeding was a more common practice among women who had not disclosed their HIV status to their families. On the other hand, women who adhered to exclusive infant feeding had disclosed their status to family members. The data suggest that the family plays a crucial role in infant feeding practices. In the context of HIV infection and the absence of disclosure, the family influences adherence to infant feeding negatively. Healthcare workers have a critical role to play in developing interventions to support HIV pregnant women to disclose in order to avoid mixed feeding practices.

Furthermore, limited knowledge of the ways through which infant feeding practices influence HIV transmission risk increased mixed feeding practices. While women who understood the transmission risk of infant feeding were able to withstand pressure from families to mix feed despite the lack of disclosure. Improving the quality of information provided to HIV-positive pregnant women during counselling will influence adherence to infant feeding and reduce mixed feeding.

These findings have implications for the PMTCT program. There is a need to develop interventions that respond to the social and cultural contexts of HIV positive mothers to improve disclosure decision making in PMTCT programs. The interventions should also take into consideration the role of the family in infant feeding practices as well as its importance in the success of the PMTCT program. Although the counselling sessions in the PMTCT program are focused on the pregnant woman, the raising of a child in the African context is family oriented which has significant implications for the PMTCT program. It is essential that the PMTCT program is refocused to include community based interventions that would educate male partners, family members and the community about infant feeding in the context of HIV infection. Furthermore, PMTCT interventions in the community should aim at improving the social context of disclosure to reduce stigma and promote HIV disclosure to significant others.
